# Molecular dynamics simulations of thermal transport in metals using a two-temperature model

**DOI:** 10.1007/s00894-025-06433-5

**Published:** 2025-07-26

**Authors:** B. Baer, D. G. Walker

**Affiliations:** 1 Interdisciplinary Materials Science, Vanderbilt University, Nashville, TN USA; 2 Mechanical Engineering, Vanderbilt University, Nashville, TN USA

**Keywords:** Two-temperature molecular dynamics, Thermal conductivity, Thermal interfaces

## Abstract

**Context:**

In classical molecular dynamics, thermal transport via electrons is typically non-existent. Therefore, thermal property determination in metals or material systems that include metals is inaccessible. We have developed a two-temperature model for use with non-equilibrium molecular dynamics to predict thermal interface resistance across metal–metal and metal–insulator interfaces. Using LAMMPS and a modified module for the diffusion of thermal energy via electrons, we systematically examine the effects of including a second transport pathway through material systems. We found that inclusion of an electronic transport pathway reduces the phonon-only thermal conductivity because of electron–phonon scattering. Moreover, the presence of electrons eliminates temperature jumps at the boundary but still admits interface resistance, which is reduced in some cases by an order of magnitude.

**Method:**

We developed a module for LAMMPS that estimates thermal transport via the diffusion equation with a specified electron thermal conductivity. The electronic energy is transferred to/from the atomic system using velocity rescaling with appropriate momentum perturbation. The atomistic motion is governed by the NiU3-EAM potential.

## Introduction

Thermal transport in metals is mediated by contributions from both phonons in the lattice and delocalized electrons. Having multiple transport pathways presents a challenge for modeling thermal transport in metals using molecular dynamics (MD) because the classical technique is limited to atomistic motion, which cannot natively account for the electronic contribution. Depending on the metal, the electronic contribution can be orders of magnitude larger than the lattice contribution. Additionally, because the effect of electrons is absent in molecular dynamics, MD potentials for metals can not be parameterized to return the correct total thermal conductivity while simultaneously reproducing other properties like stiffness that do not inherently require electron transport.

One approach to include the electronic contribution to thermal transport in MD is to use a two-temperature model (TTM), where the electrons and the lattice are treated as two separate sub-systems that are allowed to exchange energy. The TTM approach in MD has been applied to a variety of high-energy applications, such as radiation damage [1–3], laser ablation [4–8], and shock loading [9–11], where strong thermal non-equilibrium between the electrons and lattice dominates and where electron processes are sufficiently fast that separating the electronic and atomic time steps is natural.

One such TTM was proposed by Victoria and Caro [12], who devised a TTM to model electron stopping as a drag force and electron–phonon interactions with a Langevin thermostat connecting the electronic and atomic subsystems. This was implemented in the MD code DL_POLY [13] by Duffy and Rutherford [1, 14], who separated the energy exchange contributions between electronic stopping and electron–phonon scattering. This implementation was then ported to the MD code LAMMPS [15] by Phillips and Crozier [16], who improved the TTM by correcting an energy drift issue caused by the finite size and time constraints of a MD simulation. In Duffy’s implementation, the electron temperature was recalculated at every time step as if the average amount of energy had been exchanged, regardless of whether the actual energy change in the lattice was higher or lower. The LAMMPS implementation eliminated the need for the electron temperature to be fixed at the boundary to minimize energy drift, also allowing for periodic boundary conditions.

The TTM approach has been extended to simulations of steady state thermal transport [17, 18]. In particular, Wang et al. [17] applied the TTM to calculate the thermal conductivity of metals using Fourier’s law and a steady-state temperature distribution. They recovered the measured thermal conductivity ($\kappa_{\text{tot}}$) in copper and modeled thermal transport across metal-nonmetal interfaces. Because copper is dominated by electron transport (> 95% of energy is transported via electrons [19]), they and others [20, 21] chose to neglect the phonon portion of thermal transport in copper and parameterize the TTM for copper by setting the electron thermal conductivity to the experimental conductivity (that is, ${\kappa }_{e}={\kappa }_{\text{tot}}=401$ Wm^−1^ K^−1^ and ${\kappa }_{p}=0$). No simulations have been performed on metals whose lattice contribution is commensurate with the electronic contribution. Therefore, it is unclear whether the TTM is generally applicable and what the appropriate properties of the electronic system might be in the metal and at an interface when the lattice contribution is significant.

In this work, we consider the application of the TTM to a metal where the electron and lattice contributions are roughly equal. We have chosen Ni for our model material, as conductivity contributions for the electrons:lattice are split roughly 60:40. Bulk thermal conductivity is calculated, and then model metal–insulator and metal–metal interfaces are constructed to explore the effect of the TTM on interfacial transport in controlled configurations. In addition, we augment our model with a source term on the electronic side to mimic energy deposition by external processes such as inductive heating, which interacts exclusively with electronic systems in materials.

## Simulation details

A limitation of the currently available implementations of TTM within MD is that they were designed for homogeneous systems. As such, they use a single set of electronic properties for the entire simulation domain. This is sufficient for calculating transport properties in a single material, but non-homogeneous structures that include metals are critical to a wide variety of materials studies [22, 23] and applications [24] where effective transport properties of the composite system are needed. In this work, we modify the TTM implementation of Phillips and Crozier in LAMMPS to enable simulation of systems that include metal–metal and metal–insulator interfaces with varying TTM properties across the simulation domain. This approach enables us to describe the thermal transport properties of metal–metal and metal–insulator interfaces within the MD framework. Full details of the TTM may be found in their and Duffy’s publications, but a brief description of the TTM follows. Details of our modifications to the TTM are then reported.

Thermal transport within the electronic subsystem is governed by the diffusion equation;

1 $$c_{\text{vol}}\frac{\partial T_e}{\partial t}=\nabla\left(\kappa_e\nabla T_e\right)-g_p\left(T_e-T_l\right).$$

The TTM solves the finite-difference version of Eq. (1) by subdividing the simulation domain into a finite-difference grid. Each grid cell has the ensemble properties of the electrons associated with the atoms contained within the grid cell. That is, each grid cell is characterized by the volumetric electronic heat capacity (*c*_vol_), the electronic thermal conductivity (*κ*_*e*_), and the electron–ion interaction parameter (*g*_*p*_). The term on the left represents the change in stored energy within the electronic subsystem over time. The first term on the right is the diffusion term by which energy is exchanged between adjacent electron grid cells, and the second term on the right governs energy exchange between the electrons and the lattice atoms associated with each grid cell. To be clear, the diffusion equation does not model the motion of electrons but is representative of the motion of thermal energy via electrons.

The energy exchange between electrons and lattice on the atomic subsystem is accomplished by modifying the traditional MD equation of motion (Newton’s law) for an atom of mass ${m}_{i}$, velocity ${v}_{i}$, experiencing a force of ${F}_{i}$. Energy is lost through a frictional term with coefficient ${\gamma }_{i}$, and momentum is adjusted via a stochastic term $\widetilde{F}\left(t\right).$

2 $$m_i\frac{\partial v_i}{\partial t}=F_i\left(t\right)-\gamma_iv_i+\widetilde F\left(t\right),$$

where the coefficient ${\gamma }_{i}$ is related to the scattering rate ${g}_{p}$

3 $$g_p=\frac{\gamma_i3Nk_B}{Vm_i}.$$

The frictional term in Eq. (2) represents energy transferred from the lattice to the electronic subsystem, accomplished through velocity rescaling, and is related to the drag force by including the number of atoms *N* in a finite difference cell of volume *V*. The stochastic term adds momentum to the lattice from the electronic subsystem, with the force having a randomly chosen direction to mimic the effects of electron–phonon scattering. The stochastic term satisfies the fluctuation–dissipation theorem such that

4 $$\langle \widetilde{F}\left(t\right)\rangle =0$$

5 $$\langle \widetilde{F}\left(t^{\prime}\right)\cdot \widetilde{F}\left(t\right)\rangle =2{k}_{\text{B}}{T}_{e}{\gamma }_{i}\delta ({t}^{\prime}-t)$$

Equation (4) shows that momentum is conserved at any given time. Equation (5) shows how the change in momentum over time is related to the energy coupling between electrons and atomic motion. A decrease in electron temperature due to electron–phonon scattering must be balanced by an increase in energy of the lattice. Therefore, the energy added/removed from the lattice by the frictional term corresponds to the energy removed/added the electrons due to the last term on the right-hand side in Eq. (1).

We have modified the foregoing model to enable heterogeneous material properties and introduce a source term (*s*) for an external energy generation. As such, we have implemented

6 $$c_{\text{vol}}\frac{\partial T_e}{\partial t}=\nabla\left(\kappa_{\text{eff}}\nabla T_e\right)-g_p\left(T_e-T_l\right)+s,$$

where ${\kappa }_{\text{eff}}$ represents the averaged material property when adjacent grid cells have differing electronic thermal conductivity ${\kappa }_{e}$ values, and the source term is the last term in Eq. (6).

### Modification 1: Heterogeneous support

Because the original TTM was designed to work exclusively on homogeneous systems, the parameters that govern the electronic subsystem required only a single value of *κ*_*e*_, which was used for the entire simulation. We have modified the TTM by assigning electronic properties to each grid cell rather than a single value across the entire simulation. For multiple materials, we must be cognizant of the location of the grid relative to the material boundaries and the effective material properties at the cells on the interface. In our implementation, we do not require that the interface coincides with a cell boundary but have chosen to enforce this condition so that bulk properties may be used to parameterize the TTM. As such, to maintain energy conservation, thermal transport between two grid cells, indicated by the subscripts *a* and *b*, must use an effective thermal conductivity,

7 $$\kappa_{\text{eff}}=\frac{2\kappa_a\kappa_b}{\kappa_a+\kappa_b}.$$

Importantly, if ${\kappa }_{a}={\kappa }_{b}$ then ${\kappa }_{\text{eff}}={\kappa }_{a}$, so there is no loss of generalizability to homogeneous simulations.

We have also implemented support for TTM regions without electrons that can be used to represent either insulating materials (atoms but no electrons) or vacuum (no atoms and no electrons) within a multi-component simulation. In both cases, cells with no electrons do not participate in the diffusion of energy between adjacent cells and do not exchange energy with the atoms in the lattice.

### Modification 2: Addition of a source term

Some TTM implementations have included a source term in the electronic diffusion equation so that energy can be added directly to the electronic subsystem. Adding energy to the electrons can be used to model energy absorption from interactions such as the aforementioned laser heating and joule heating [25, 26] or to serve as a thermostat for the electronic subsystem [27]. These implementations often make use of domain specific parameterizations that limit transferability, such as defining laser spot sizes or voltages across the simulation cell. We have chosen instead to implement a generalized source term.

8 $$s=P\eta \left(r\right)$$

where $P$ is the power applied across the simulation domain, while $\eta \left(r\right)$ is used as an absorption efficiency that varies spatially so that our ability to model multiple materials is preserved. It should be noted that $P$ can be given a time dependence and implemented as $P\left(t\right)$ but for the purposes of steady-state thermal transport we have chosen to implemented $P$ as a constant.

## Results

### Material selection

We have chosen nickel as our model material. Nickel is an important metal both for its metallurgical properties [28, 29], as well as a catalytically active transition metal [30, 31]. Particularly in its role as a catalyst in endothermic reactions, the ability to accurately model thermal transport could prove useful.

DFT results for Ni have shown that the thermal conductivity contributions of electrons and phonons are of the same order of magnitude. At 300 K, ${\kappa }_{\text{tot}}$=91 Wm^−1^ K^−1^, which is partitioned into a phonon contribution of ${\kappa }_{p}$ = 19 Wm^−1^ K^−1^ and an electronic contribution of ${\kappa }_{e}$ = 72 Wm^−1^ K^−1^ [19]. This makes Ni an interesting test case for the TTM because the TTM must work in tandem with the MD of the lattice to return the correct total thermal conductivity. In MD, the ${\kappa }_{p}$ contribution is determined by the parametrization of the chosen potential; therefore, the ${\kappa }_{e}$ used in the TTM can be used as a free parameter to reproduce the correct total thermal conductivity.

The thermal conductivity of bulk Ni using both traditional MD and TTM-MD was computed to demonstrate a calculation using the TTM where phonons have a non-trivial contribution. Before a suitable potential was selected, a brief survey of available potentials for nickel were examined. Total thermal conductivity estimates for metals derived from EAM potentials are grossly inaccurate for reproducing the total thermal conductivity as summarized in Table 1 due to the lack of electronic effects built into the potentials. Understandably, the potentials listed in Table 1 were optimized for properties other than thermal conductivity that do not require electronic effects, so a good match with total thermal conductivity is not expected. Ultimately the NiU3 EAM potential of Foiles et al. [32] was selected for Ni–Ni interactions in the MD because EAM potentials are commonly used for MD simulations of metals, and the NiU3 potential does an admirable job at reproducing the expected phonon thermal conductivity of nickel. In fact, the results will show the lattice thermal conductivity to be 13% less than the DFT calculated value from the literature.

**Table 1:** Thermal conductivity of various EAM potentials

Potential name (LAMMPS style)	Thermal conductivity (Wm^−1^K^−1^)
NiU3 (eam)[32]	**23.83**
Zhou (eam/alloy) [33]	30.41
SMF7 (eam) [34]	25.66
NiAlH (eam/fs) [35]	26.97
Ni99 (eam/alloy) [36]	21.43
Mendelev (eam/fs) [37]	66.24

### Thermal conductivity of bulk nickel

**Fig. 1:**
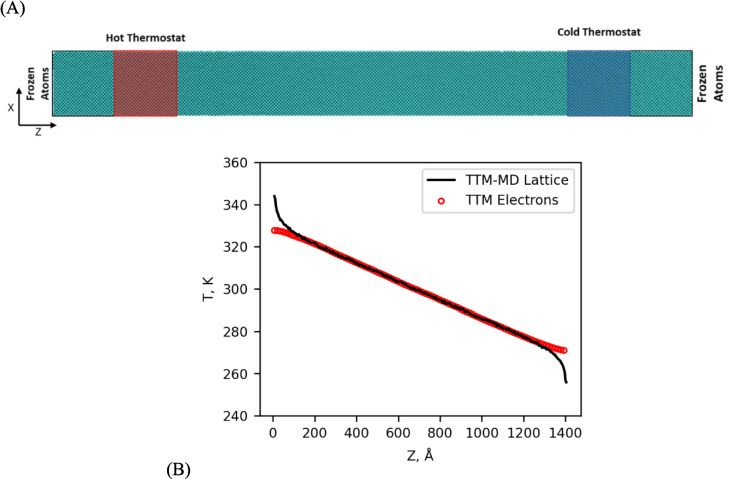
(**A**) A depiction of a typical NEMD simulation, rendered using VMD [38]. The outer layer of atoms is frozen to break periodicity in the direction of transport. The next layer of atoms at either end is thermostated to create a temperature gradient across the simulation. (**B**) An example temperature gradient using TTM-MD. Note the electron–phonon equilibrium in the central region of the simulation and the non-equilibrium near the thermostated regions

To calculate thermal conductivity, we make use of the non-equilibrium MD (NEMD) method, sometimes called the direct method. The general characteristics of this method are illustrated in Fig. 1, and apply to both MD and TTM-MD. A simulation domain is constructed with an elongated direction in which transport will be measured (Fig. 1A); transport in the other two directions is restricted, so the lengths are limited and periodic boundaries are sufficient. The outermost layers of atoms are frozen, which means they are excluded from time integration within MD. Consequently, they never move, and the boundaries serve to break the periodicity in the direction of transport. The next layer of atoms at either end of the simulation is thermostated at temperatures ${T}_{\text{hot}}$ and ${T}_{\text{cold}}$ to establish a steady-state temperature gradient (as in Fig. 1B) across the simulation. Fourier’s law was used to calculate the thermal conductivity based on the heat flux (*J*) and temperature gradient ($\nabla T$).

9 $$J=\kappa_{\text{tot}}\nabla T,$$

The heat flux was calculated as

10 $$J=\frac{Q_{\text{hot}}-Q_{\text{cold}}}{2tA},$$

where ${Q}_{\text{hot}}$ and ${Q}_{\text{cold}}$ are the cumulative energy added and removed by the hot and cold thermostats over the duration of the simulation, $A$ is the cross-sectional area of the simulation and $t$ is the time over which the simulation was averaged. The temperature gradient is given as the imposed temperature difference divided by the length of the un-thermostated region.

**Fig. 2:**
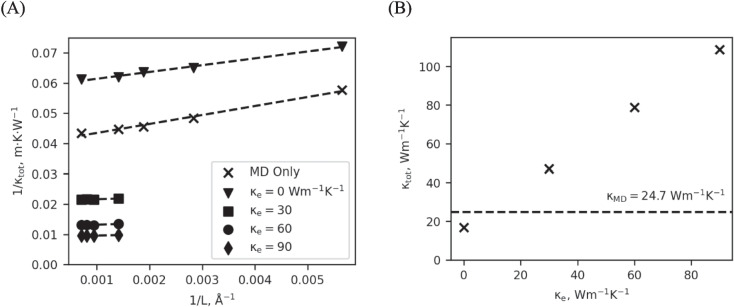
(**A**) Inverse thermal conductivity vs. inverse length. The y-intercept of the linear regression is used to estimate the bulk thermal conductivity (as L → ∞). (**B**) The total thermal conductivity is the extrapolated value from (A) shown for the specified electronic conductivity. The value from MD alone is indicated by the dashed line

However, traditional MD simulations suffer from finite size effects because the longest wavelength phonon possible is determined by the size of the simulation. Therefore, short lengths will usually result in thermal conductivities lower than longer simulations as the population of long-wavelength phonons is artificially limited. To address this shortcoming, we use the method proposed by Schelling et al. [39]. They propose that thermal conductivity be determined for a variety of system lengths and then plotted as $1/{\kappa }_{\text{tot}}$ vs. $1/L$, which allows the bulk conductivity to be extrapolated to lengths far beyond the finite size effects (see Fig. 2A). The y-intercept of the linear fit represents the conductivity of a system of infinite length (i.e., the bulk conductivity). Following this procedure for calculation of bulk thermal conductivity, systems of various lengths were prepared at $20\times 20\times N$ unit cells, with $N$ varying from 50 to 400 unit cells.

Each simulation length was brought to 300 K under NVT over 50 ps, and then, the system was relaxed under NPT at 0 atm and 300 K for a further 50 ps with a time step of 0.5 fs. The final pressure relaxation is required to remove stress from thermal expansion. After pressure relaxation, NEMD conditions were established with the Z-direction chosen as the direction of transport. We utilized Langevin thermostats [40] set to 350/250 K for the hot/cold thermostat respectively and run until a steady-state temperature gradient was established. These thermostat temperatures were chosen so that the simulation would be centered around 300 K and room temperature properties could be used. The time to reach steady state took between 0.4 and 1 ns, depending on system length. The simulations were then run at steady state to collect time averaged data on the heat flux and temperature gradient for another 0.4–0.9 ns depending on system length. The temperature gradient was obtained by fitting the middle half of the simulated temperature distribution in order to capture the linear region that is unaffected by the local effects of the thermostats at either end of the simulation.

To calculate the total thermal conductivity with TTM-MD, the MD simulation procedure described above was repeated while simultaneously solving for thermal transport via the electronic system using the TTM-MD for a specified ${\kappa }_{e}$. The TTM FD grid used to solve the diffusion equation was sized at 1 × 1 × M, where M was set to the thickness of one unit cell for the TTM cells in the direction of transport, giving 1600 atoms per FD cell across all TTM-MD simulations. We do not expect electron temperature gradients orthogonal to the direction of transport, so these directions were not discretized. TTM parameters for Ni were ${c}_{\text{vol}}$=0.170 Jcm^−3^ K^−1^ [41] and ${g}_{p}$=44 × 10^16^ Wm^−3^ K^−1^ [42]. The electron thermal conductivity, ${\kappa }_{e}$, was a free parameter varying between 0 and 90 Wm^−1^ K^−1^. The temperature dependence of these properties was ignored to a reasonable approximation in this study because the central region where data were collected was within ± 20 K of properties taken at 300 K. As such, the variation in electronic thermal conductivity (arguable the most sensitive of the three parameters) is a maximum of 3% across the temperature range at 300 K [43].

TTM-MD simulations were equilibrated for an additional 300 ps with the TTM enabled. The higher thermal conductivity from electronic transport allowed the TTM-MD simulations to establish the new steady-state in less time than the MD simulations. TTM-MD simulations were then run for 300 ps at steady-state to collect time averaged data on the heat flux and temperature gradient.

The application of Fourier’s law (Eq. 9) to calculate thermal conductivity with TTM-MD is confounded by the addition of a second temperature gradient inherent to the electronic subsystem within the TTM. However, if the hot and cold thermostats are separated by a distance sufficient to allow the electrons and lattice to reach local thermodynamic equilibrium (i.e., ${T}_{e}={T}_{l}$), then the temperature gradient is unambiguous. Wang et al. [17] defined the length (${L}_{ne}$) that the lattice and electrons were out of thermal equilibrium near an interface as

11 $$L_{ne}\approx\frac3{\sqrt{g_p\left(\frac1{\kappa_p}+\frac1{\kappa_e}\right)}}.$$

Although this expression was derived for an interface, it works equally well to define the non-equilibrium length caused by the thermostat at the edges of a NEMD simulation. Now, as long as the simulation domain is sufficiently longer than twice this length (due to a thermostat being present on both sides of the simulation), then $\nabla {T}_{p}=\nabla {T}_{e}$ in the middle region of the simulation where the gradient is measured. The non-equilibrium length for Ni with our TTM parameters is $L_{ne}\approx200\mathring{A}$, so TTM-MD simulations were limited to lengths greater than 200 unit cells (~ 700 Å) to ensure a sufficiently large linear region in the center where electron–phonon equilibrium is established. This condition limits TTM-MD simulations to rather large lengths, and increases the associated computational expense. Therefore, we devised a strategy to incorporate systems shorter than 200 unit cells, which will be examined in the next section.

As expected, the thermal conductivity of TTM-MD materials with an electronic contribution to transport is higher than thermal conductivity predicted for the lattice alone. We tested the naive assumption that TTM-MD with ${\kappa }_{e}$= 0 Wm^−1^ k^−1^ would return a total thermal conductivity equal to that of MD alone. Instead, we find that the thermal conductivity of TTM-MD with ${\kappa }_{e}$= 0 Wm^−1^ k^−1^ is lower than that of MD by 30%, indicating that the TTM’s mechanism of implementing energy exchange between the two subsystems via velocity rescaling with a stochastic redistribution of momentum has a reducing effect on phonon thermal transport. This effect, though, is not simply a computational artifact. Yuan et al. [44] estimated that with the inclusion of electronic effects, the mean free path for phonons in Ni at 300 K is reduced to ~ 1 nm due to electron–phonon scattering. Similarly, calculations by Gall [45] predicted a mean free path for electron–phonon scattering of ~ 6 nm. The presence of electrons and subsequent exchange of energy between electrons and phonons, even when the two systems are in equilibrium, introduces a scattering mechanism which decreases the mean free path of phonons. Therefore, the lattice thermal conductivity with electron scattering should be less than that calculated by a MD-only simulation. Furthermore, we observe that the TTM-MD results for ${\kappa }_{e}$=30–90 Wm^−1^ K^−1^ show very little length dependence. The length of our TTM-MD simulations is many times larger than both the Yuan and the Gall mean free path estimates. Therefore, our simulation lengths are within the bulk regime for thermal transport. Thus, the absence of the total thermal conductivity’s length dependence in TTM-MD is an appropriate consequence of including electronic effects in MD.

**Fig. 3:**
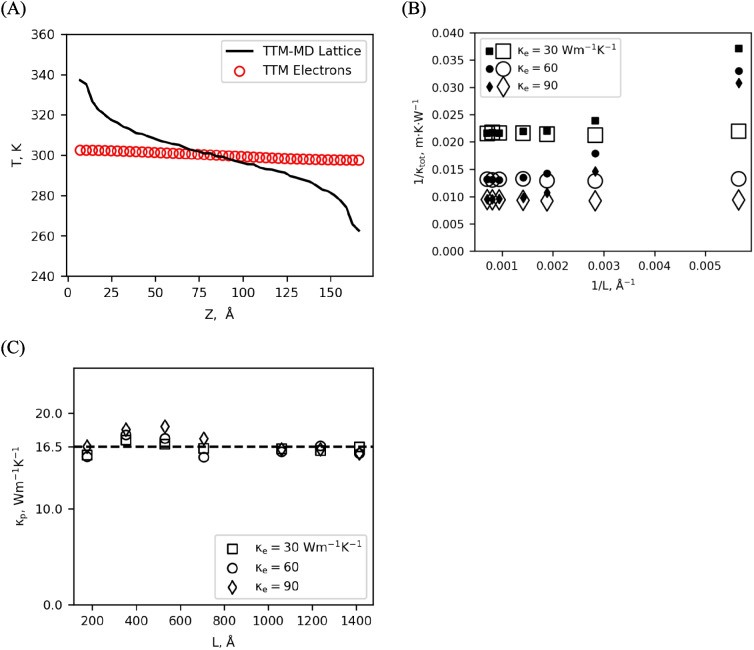
(**A**) For short length TTM-MD simulations (50 unit cells shown), electron–phonon equilibrium cannot be established even at steady state. (**B**) If short systems are analyzed using Fourier’s law (Eq. (9), closed marks), the length dependence of *κ* becomes non-linear. If they are instead analyzed using the composite Fourier’s law (Eq. (12), open marks), linearity is restored. However, even with shorter systems, ${\kappa }_{\text{tot}}$ still has no length dependence in TTM-MD. (**C**) The phonon thermal conductivity calculated under TTM-MD is independent of both system length and TTM parameterization for ${\kappa }_{e}$

By treating the electronic thermal conductivity as a free parameter and comparing results from simulations using different values of ${\kappa }_{e}$, we learn how the two systems (lattice and electronic) interact. The relationship between ${\kappa }_{e}$ and ${\kappa }_{\text{tot}}$ is linear, as can be seen in Fig. 2B, which indicates that the total thermal conductivity is a sum of the electronic and lattice contributions and ${\kappa }_{p}$ is constant. However, the lattice contribution in this sum is not the lattice thermal conductivity from a MD-only simulation. Instead, the lattice thermal conductivity under the TTM is obtained from the TTM-MD simulation with ${\kappa }_{e}$= 0. The electron–phonon interactions of the TTM lower the lattice contribution to the thermal conductivity across all values of ${\kappa }_{e}$, and this reduction must be considered when selecting ${\kappa }_{e}$ to return the correct ${\kappa }_{\text{tot}}$. Therefore, it is not sufficient to obtain the lattice conductivity from an MD-only simulation and add the electronic conductivity; the coupled system must be run under TTM-MD to include the effects of electron phonon scattering on the lattice component. Furthermore, when electronic transport is enabled (${\kappa }_{e}>0$), the lattice conductivity is independent of both device length and electronic thermal conductivity (Fig. 3C). This feature is also important because thermal conductivity of electrons is not a quantity that is often measured experimentally and can be difficult to pin down from the literature. Therefore, we suggest that ${\kappa }_{e}$ more often be the deduced parameter from the known total thermal conductivity of materials and calculated lattice thermal conductivity at ${\kappa }_{e}$=0.

### Using short simulation lengths in TTM-MD

As mentioned previously, meeting the condition of simulation length sufficiently above 

$2{L}_{ne}$ requires TTM-MD systems greater than ~ 200 unit cells in length based on our chosen properties for Ni. Larger simulations are obviously more costly, and it would be beneficial to obtain results from smaller systems as used in the traditional MD runs that range in length from 50–200 unit cells.

In the case of simulations with short lengths, there is strong non-equilibrium between the electrons and phonons through the entire simulation, as seen in Fig. 3A for a 50 unit cell simulation. This causes a breakdown of Fourier’s law, because the condition of $\nabla {T}_{e}=\nabla {T}_{p}$ is not met. When these smaller systems are used, an incorrect total thermal conductivity is obtained from a naive implementation of Fourier’s law, as seen in Fig. 3B with the solid markers. In smaller simulations, $\nabla {T}_{e}$ will be less than $\nabla {T}_{p}$ as the electronic thermal transport moves energy from one thermostat to the other before it has sufficient time to exchange with the lattice. This leads to an increase in the heat flux without a corresponding change in the temperature gradient of the lattice, leading to an overestimation of ${\kappa }_{p}$ under Eq. (9) in conditions of non-equilibrium. The degree of this overestimation depends on the degree of non-equilibrium, which itself depends on the total simulation length, ultimately resulting in a non-linear relationship between 1/${\kappa }_{\text{tot}}$ and 1/L.

For short simulation lengths where local thermodynamic equilibrium cannot be established, we instead use a composite Fourier’s law for metals;

12 $$J=\kappa_p\nabla T_p+\kappa_e\nabla T_e,$$

where the electronic and lattice contributions to thermal conductivity are considered separately. This allows us to take the electron temperature gradient in the middle half of the simulation in the same manner as the lattice temperature gradient and use the electronic thermal conductivity value that was an input parameter to the TTM to calculate the lattice thermal conductivity. Importantly, Eq. (12) will reduce to the typical form of Fourier’s law as in Eq. (9) if $\nabla {T}_{e}=\nabla {T}_{p}$, with ${\kappa }_{\text{tot}}={\kappa }_{e}+{\kappa }_{p}$. This is essentially the condition that is enforced by the minimum system length requirement. Figure 3B shows the results of using both Eq. (9) (solid marks) and Eq. (12) (hollow marks) for various values of ${\kappa }_{e}$. When the composite formulation of Fourier’s law is used, simulations less than 200 unit cells produce consistent results with systems greater than 200 unit cells, and a linear relationship between $1/{\kappa }_{\text{tot}}$ and $1/L$ is obtained.

Interestingly, the inclusion of less than 200 unit cell systems still indicates that there is no simulation length dependence in either the phonon thermal conductivity or the total thermal conductivity. Figure 3C shows a constant ~ 16.5 Wm^−1^ K^−1^ phonon thermal conductivity value across all tested simulation sizes. This result is in line with the bulk thermal conductivity value obtained from fitting the length dependence of the simulations run with ${\kappa }_{e}=0$, and indicates that the lack of length dependence observed in Fig. 2A for TTM-MD simulations extends down to systems of at least 50 unit cells (~ 525 Å) in length.

### Inductive heating via a TTM source term

So far we have considered systems where the internal generation is negligible. However, some TTM-MD applications require a source term by which energy can be added to the electronic subsystem. For example, laser-metal interactions involve the addition of thermal energy to electrons followed by equilibration with the lattice through electron–phonon scattering. In previous treatments, these source terms are primarily intended to capture the transient response to laser heating and are not suitable for modeling steady-state thermal transport. We instead extend our model to use a source term designed to mimic inductive heating. That is, thermal energy is added to the electrically conductive material using a high frequency alternating current in a coil around the target material. Inductive heating techniques have been used in applications such as heat-treating metals [46], reactor heating [47], and nanoparticle synthesis [48, 49].

**Fig. 4:**
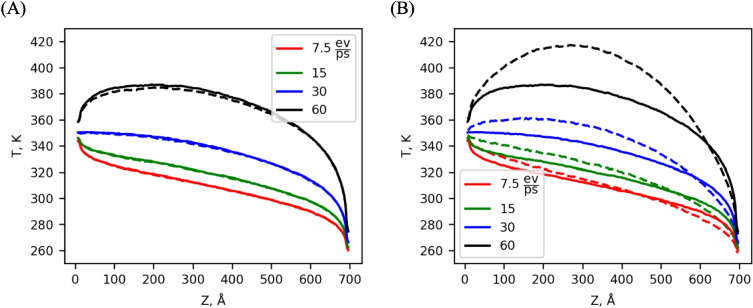
Lattice temperature under TTM heating (dashed lines) and lattice heating (solid lines) for various generation rates. (**A**) Normal electron properties for Ni. (**B**) The effect of weakening electron–phonon coupling to 1/10th of its previous value

We have implemented electronic heating as a source term in our TTM as Eq. (8) and compared it against adding energy directly to the lattice with a global thermostat. The previous NEMD simulations described above serve as the starting point for the simulations with generation. The chosen simulation is 300 unit cells in length, with hot and cold thermostats at each end set to 350/250 K respectively. The TTM is parameterized for Ni with *κ*_*e*_ = 60 Wm^−1^ K^−1^ and participates in thermal transport in all simulations, regardless of whether we heat the electronic system or the lattice directly. For simulations using TTM heating (Fig. 4, dashed lines), we applied a variety of generation rates distributed uniformly across the electronic cells in the TTM. We also prepared simulations where the same generation was added directly to the lattice (termed lattice heating), distributed evenly across the atoms (Fig. 4, solid lines) in the simulation via velocity rescaling.

A range of generation rates were tested based on the generation rate in the original NEMD simulation (15 eVps^−1^). These generation rates ranged from half the original generation rate (7.5 eVps^−1^) to four times (60 eVps^−1^). Adding energy volumetrically instead of just at the edges of the simulation changed the temperature profile of the simulations from its original linear shape for heating rates of 7.5 and 15 eVps^−1^, to a parabolic-ish shape for 45 eVps^−1^ and 60 eVps^−1^ with the degree of change dependent on the strength of the inductive heating. The temperature distributions are perfectly analogous to the bulk solutions to the diffusion equation, with (parabolic) and without (linear) uniform generation, although the non-equilibrium present at the thermostats can not be captured with continuum simulations. The temperature setpoints of the thermostats remained unchanged, which caused the hot thermostat to add less heat as the inductive heating was increased and eventually change sign to remove energy from the system for ${P}_{\text{ind}}$= 60 eVps^−1^.

As seen in Fig. 4A, adding energy to the electronic subsystem via TTM heating or adding energy directly to the lattice via velocity rescaling produces marginally the same results in the temperature distribution for our chosen Ni properties. Some small deviation is apparent in the 60 eVps^−1^ case as the maximum temperature of the system under TTM heating is slightly lower than the maximum temperature with velocity rescaling. This trend is expected as the energy deposited directly into the electrons under TTM heating is immediately able to diffuse to adjacent electron cells towards the thermostats at the edges where non-equilibrium allows greater energy transfer between the systems. While energy added to the lattice via velocity rescaling is limited by ${g}_{p}$ to enter the electronic subsystem for transport.

To further explore the effect of electron phonon non-equilibrium, an additional set of TTM heating simulations were performed where the value of ${g}_{p}$ was reduced to one tenth of its previous value (dashed lines in Fig. 4B). If the energy transfer between the two systems is reduced, we expect the electronic heating system to result in lower lattice temperatures. These simulations produced a markedly different temperature profile between TTM heating and atomic heating, demonstrating that there are cases where electronic heating is not equivalent to atomic heating. The disparities become more pronounced for either higher generation rates or weaker interaction between electrons and phonons.

### Modeling interfaces with TTM-MD

To test the TTM with interfaces, we have constructed a toy model where all atoms use the same interatomic potential. Although, this can be considered non-physical, using the same potential instead of two different materials and their respective potentials allows us to modify the interface systematically by changing one parameter at a time, while also not requiring a parameterization or mixing of potentials between the two materials comprising the interface.

All atomic interactions are governed by the NiU3 EAM potential used in previous sections. The simulation domain is comprised of 20 × 20 × 400 FCC unit cells following the same NEMD procedure as before. To create an interface, the simulation is split in half along the Z axis, with MD and TTM-MD properties assigned independently to each half. Five interface cases were tested, with a summary of the conditions for each interface outlined in Table 2.

**Table 2:** Interface cases

		Material 1		Material 2	
	Interface type	Atomic mass (amu)	***κ***_***e***_ (Wm^−1^ k^−1^)	Atomic mass (amu)	***κ***_***e***_ (Wm^−1^ k^−1^)
Case 1	Insulator–insulator	58.71	-	117.39	-
Case 2	Metal–insulator	58.71	74	117.39	-
Case 3	Metal–metal	58.71	74	117.39	52.33
Case 4	Metal–insulator	58.71	74	58.71	-
Case 5	Metal–metal	58.71	74	58.71	52.33

Since we used the same potential for all atoms, an interface was introduced by changing the atomic mass to modify the lattice conductivity. From kinetic arguments, the phonon conductivity should scale with the group velocity as

13 $$\kappa_p=\frac13cv_gl,$$

where $c$ is the phonon specific heat, ${v}_{g}$ is the phonon group velocity and $l$ is the phonon mean free path. The group velocity (also approximately the sound speed of a material) is found from the derivative of the phonon dispersion and can be modified by changing the frequency of phonon modes. From a 1D harmonic oscillator

14 $$\omega=\sqrt{\frac km}.$$

If the spring constant, $k$, remains unchanged through the use of the same potential, we can expect to reduce ${\kappa }_{p}$ by a factor of $\sqrt{2}$ by doubling the atomic mass.

#### Cases 1–3: ${m}_{1}\ne {m}_{2}$

**Fig. 5:**
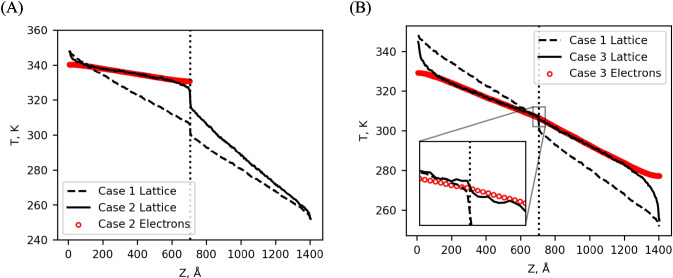
(**A**) Cases 1 and 2 are being compared. The action of the TTM only on the left side increases the temperature on that side, leading to an increase in $\Delta {\text{T}}_{\text{i}}$. The location of the interface is indicated by the dotted line. (**B**) Cases 1 and 3 are being compared. Even though the mass difference is the same as case 1, the TTM significantly reduces $\Delta {\text{T}}_{\text{i}}$ in case 3 because it is active on both sides of the interface. A small $\Delta {\text{T}}_{\text{i}}$ can be observed in the inset

The first three cases share the commonality that the mass of atoms on one side of the simulation has been doubled to create an acoustic impedance mismatch. This presumably results in a temperature jump at the interface. The difference between the simulations has to do with the addition of the TTM for the two regions of atoms. The first three simulations to consider are all insulator (case 1), half metal/half insulator (case 2), and all metal (case 3). In case 1 (Fig. 5, dashed line), the TTM is not enabled on either side. This represents an insulator–insulator interface and serves as a baseline for comparison with subsequent TTM-MD simulations. Case 2 (Fig. 5A, solid line) is similar to case 1 except that the TTM is enabled on the left side, representing a metal–insulator interface. Finally, case 3 (Fig. 5B, solid line) extends the TTM across the entire simulation to represent a metal–metal interface. To match the ratio of electronic to lattice thermal conductivities across the whole simulation, TTM cells on the side of the simulation with the doubled atomic mass had ${\kappa }_{e}$ reduced by a factor of $\sqrt{2}$ so that neither the lattice nor electronic pathways would dominate either side of the simulation. Consequently, the total conductivity of the heavy atom side is reduced by a factor of $\sqrt{2}$ compared to the normal atom side.

The interface resistance (${R}_{i}$) is calculated from the steady-state simulation by observing the temperature drop across the interface ($\Delta {T}_{i}$) and the heat flux ($J$) measured from the simulation as

15 $$R_i=\frac{\Delta T_i}J.$$

The interface resistance results for each case are listed in Table 3, along with the associated temperature drop and heat current.

**Table 3:** Interface resistances for cases 1–3

System	${R}_{i}$ (mm^2^ KW^−1^)	${R}_{L}$ (mm^2^ KW^−1^)	$\Delta {T}_{i}$ K)	Heat current (W)
Case 1	4.40 × 10^−4^	7.05 × 10^−4^	4.98	5.66 × 10^−7^
Case 2	5.03 × 10^−4^	1.19 × 10^−3^	7.19	7.15 × 10^−7^
Case 3	4.70 × 10^−5^	4.68 × 10^−6^	1.43	1.52 × 10^−6^

When comparing the interface resistance between case 1 and case 2, we find that the metal–insulator resistance is 15% larger than the insulator-insulator resistance. The TTM cannot act across the interface, so all interfacial thermal transport is phonon-mediated. Thus, the TTM increases thermal transport in the metal side, which decreases the temperature gradient across the metal side of the simulation, but the phonon properties of the interface remain largely unaltered. The modest increase in interface resistance for the metal–insulator interface is driven by the larger $\Delta {T}_{i}$ as a result of the TTM increasing the heat through the system. While it is beyond the scope of our current implementation, work on a TTM with non-localized (i.e., across the interface) scattering has been developed by Lu et al. [50].

When comparing cases 1 and 3, it is apparent that the interfacial thermal transport decreases by an order of magnitude by the action of the TTM. In case 3, the interface is no longer mediated by phonons only. Instead, the electronic system is allowed to transmit thermal energy across the interface. Allowing a non-phonon-mediated pathway across the interface reduces the ${R}_{i}$ significantly when compared to either system involving an insulator (cases 1 and 2) because the heat current is nearly tripled and the $\Delta {T}_{i}$ is very small at only 1.4 K (Fig. 5B, inset).

#### Cases 4 and 5: ${m}_{1}={m}_{2}$

**Fig. 6:**
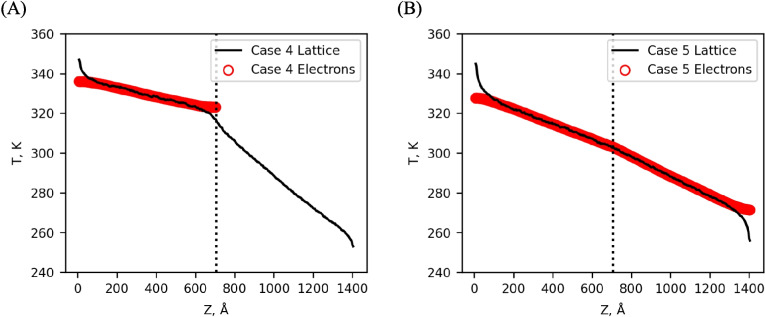
(**A**) Case 4. Without a mass difference across the interface, the $\Delta {T}_{i}$ is similar to the temperature gradient in either material. The effect of the interface can still be observed in the non-linearity of the temperature gradient near the interface. (**B**) Case 5. Like case 4, the lack of a mass difference at the interface leads to a small $\Delta {T}_{i}$, especially combined with the additional effect of the TTM acting on both sides of the interface

Two additional cases in our toy model where there is no mass difference between atoms on either side of the simulation are studied to understand the action of the TTM when a temperature jump is not present in the lattice system. If the atomic mass is constant, then the only difference in the simulation can be the presence of the TTM. This configuration reduces to two cases that have already been simulated (i.e., the insulator–insulator case and metal–metal case in section B above), which we call bulk simulations in Table 4. These simulations are outlined in previous sections and have no interface resistance by definition. Case 4 (Fig. 6A) is the metal–insulator case, similar to case 2 with the mass difference removed. Similarly, case 5 (Fig. 6B) is based on case 3 with the mass difference removed. In both cases, the interface is defined by a change in the electronic thermal conductivity alone. The interface resistances for these cases are shown in Table 4.

**Table 4:** Interface resistances

System	${R}_{i}$ (mm^2^ KW × ^1^)	${R}_{L}$ (mm^2^ KW × ^1^)	$\Delta {T}_{i}$ (K)		Heat current (W)
Bulk insulator	2.03 × 10^−5^	− 1.13 × 10^−5^		0.29	7.14 × 10^−7^
Bulk metal	1.55 × 10^−5^	− 4.44 × 10^−6^		0.564	1.82 × 10^−6^
Case 4	3.25 × 10^−5^	4.21 × 10^−4^		0.654	1.01 × 10^−6^
Case 5	4.42 × 10^−7^	2.36 × 10^−5^		0.015	1.69 × 10^−6^

Even though there is no interface in a bulk material, the discretization of the MD simulation allows for calculating the temperature difference between adjacent cells that can be used to calculate an interface resistance. However, since there is no real interface, the choice $\Delta {T}_{i}$ is somewhat arbitrary. To reduce the impact of noise, $\Delta {T}_{i}$ for the bulk systems was instead calculated using the temperature gradient as

16 $$\Delta {T}_{i}=\frac{\partial T}{\partial z}\Delta z$$

where $\Delta z$ is the bin width. This effectively gives the average $\Delta {T}_{i}$, and the interface resistance was then calculated using this temperature difference.

For both cases 4 and 5, $\Delta {T}_{i}$ is on the order of the bulk systems, and the interface is characterized as a discontinuity in the temperature distribution. As expected, a larger $\Delta {T}_{i}$ is observed for case 4 than case 5, with the addition of the electronic pathway across the interface reducing the temperature drop, which is the same trend we observed comparing cases 2 and 3. The $\Delta {T}_{i}$ in both case 4 and case 5 is the same order as the bulk simulations, which suggests that a temperature jump is due primarily to lattice impedance mismatch and not changes in electronic properties. In particular, case 5 (metal–metal interface) indicates that an electronic interface is not sufficient to introduce a measurable $\Delta {T}_{i}$ into the lattice.

**Fig. 7:**
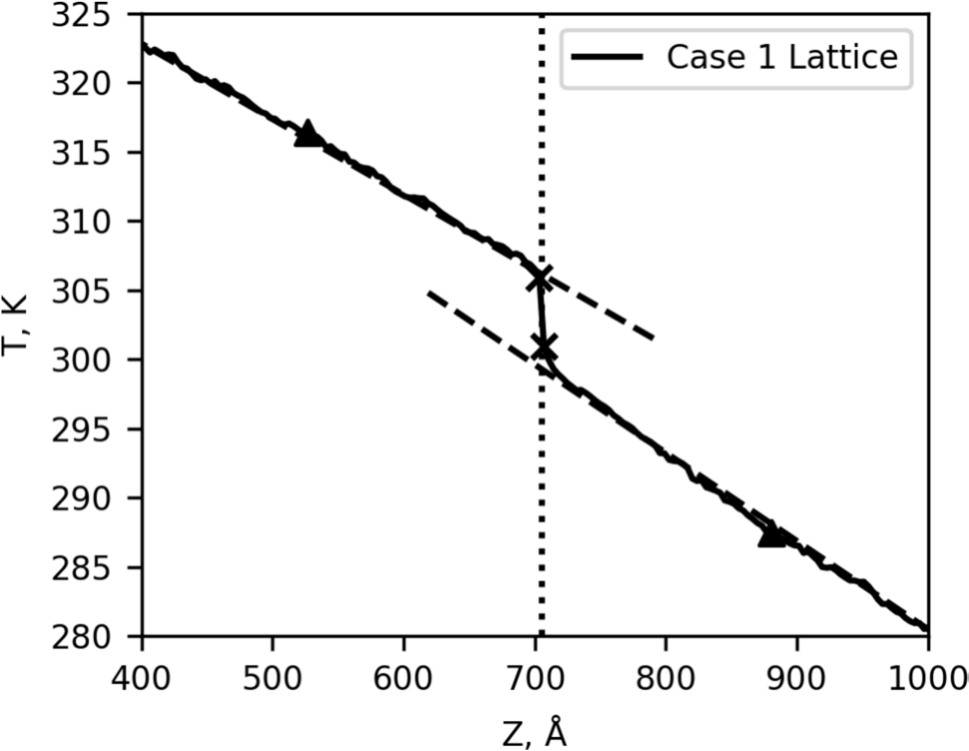
The interface region for case 1. $\Delta {T}_{i}$ is taken from the X markers to calculate ${R}_{i}$ with Eq. 15. To calculate ${R}_{L}$ using Eq. (17), $\Delta {T}_{L}$ is taken from the two triangle markers a distance $\text{L}$ from the interface in either direction. The dashed lines indicate the temperature gradients used to calculate the thermal conductivity for each material

Calculating the interface resistance by Eq. (15) is natural when the temperature difference is large. However, this method neglects the nonlinear temperature gradient that develops because of the non-equilibrium at the interface. For cases 4 and 5, because there is no mass difference, $\Delta {T}_{i}$ is on the order of the bulk systems, and the dominant effect of the interface is the discontinuity of the temperature distribution near the interface. To capture this non-equilibrium in the interface resistance, we have also calculated the interface resistance as ${R}_{L}$, which is shown in Tables 3 and 4 along side ${R}_{i}$, using the series resistance.

17 $$\frac{\Delta T_L}J=\frac L{\kappa_1}+\frac L{\kappa_2}+R_{L,}$$

where $\Delta {T}_{L}$ is the temperature difference between two points within the linear region for each material (denoted by the triangles) which were chosen to lie a distance $L\approx$ 175 Å away from the interface. ${\kappa }_{1}$ and ${\kappa }_{2}$ are the thermal conductivities of material 1 and material 2 respectively. By taking the temperature difference away from the interface (denoted by the x marks in Fig. 7), we can capture the non-equilibrium around the interface as part of the interface resistance. More importantly, we are not relying on an arbitrary and subjective definition of the temperature jump at the interface.

For cases 1 and 2, ${R}_{L}$ is roughly twice that of ${R}_{i}$ as Eq. 17 captures the additional resistance due to the non-equilibrium near the interface. Case 3 shows an order of magnitude difference between the two methods, but we suspect this difference is not meaningful as both methods show case 3 having significantly lower resistance than cases 1 or 2, indicating that we might be seeing the effects of noise in the lattice temperature or heat current. The results for the bulk systems support this, as they have resistance values on the same order of magnitude but have flipped to a negative value. Because the resistance for each of these bulk systems should be 0 by definition, the negative value lends further support that we are sampling the noise about zero.

For cases 4 and 5, ${R}_{L}$ is roughly ten times and one hundred times ${R}_{i}$ respectively. These cases, where there is no mass difference to drive a large $\Delta {T}_{i}$ show a significant difference between the two methods because Eq. (15) cannot capture the nonlinear temperature gradient outside the immediate interface cells.

Ultimately, both methods reinforce that having the TTM enabled on both sides of the interface will significantly reduce the interface resistance by providing a non-phonon-mediated pathway for energy to cross the interface. Because there is no mechanism in the diffusion equation to have a temperature drop in the electronic subsystem across the interface, the TTM will act to minimize the $\Delta {T}_{i}$ in the atoms as it drives towards equilibration between the electrons and lattice. Similarly, the electrons do not exhibit non-linear temperature gradients near the interface, so the TTM will also reduce the non-linearity of the temperature gradient at the interface when the TTM acts on both sides of the interface.

## Conclusions

In this work, we have used TTM-MD simulations to couple electron and phonon thermal transport in simulations of bulk metals and across idealized metal–insulator and metal–metal interfaces. The addition of electronic thermal transport to multi-component MD simulations allows for a more physical basis for the modeling of interfacial thermal resistance when compared to conventional MD simulations. We have demonstrated how to select appropriate TTM parameters for bulk metal simulations to reproduce experimental total thermal conductivities as well as highlighted the importance of including the effects of electron–phonon scattering when comparing the phonon contributions to thermal conductivity between MD and TTM-MD simulations. To match the thermal conductivity split between electrons and phonons as calculated by DFT, it is advisable to select a potential that produces a greater conductivity than that of the target phonon conductivity from MD without the TTM to account for the reduction in phonon transport caused by the TTM. The TTM does not simply add an additional pathway for thermal transport; electron–phonon scattering also modifies the phonon transport in the MD, and this effect should not be neglected.

This feature of the confounding feature of the TTM has been corroborated by Wu and Luo [51]. They found that increasing the electron–phonon coupling strength also increased the interfacial transport at a metal–insulator interface. The transport at such an interface is phonon mediated, but the scattering effects of the TTM help redistribute phonon energy from modes that cannot cross the interface into modes that can cross the interface. Their findings reinforce the importance of ${g}_{p}$ as a critical parameter because it not only affects energy exchange between the two systems but also modifies the phonons in the MD.

To enable the features of the TTM as examined in this work, we have written a fix (in the language of the LAMMPS software package) that allows for non-homogeneous input parameters in the TTM where electronic properties (${c}_{\text{vol}}$, ${\kappa }_{e}$, and ${g}_{p}$) can be spatially defined within the TTM. This implementation of the TTM shares the general weakness of the method in that it is highly dependent on accurate physical properties as input parameters, and these properties are difficult to determine experimentally as evidenced by the wide range of reported values. However, the computations proceed with unmeasurable cost in computational resources because the diffusion equation is discretized over a small number of TTM cells and the liner algebra calculation is scant compared to the computational work of the MD to compute forces between hundreds of thousands to millions of atoms.

Two additional areas of improvement exist for our implementation because of the presence of interfaces. The first is that no attempt was made to include the effects of electron–phonon scattering across the interface. Work in this area has been done [50] and could potentially be integrated with an updated version of our TTM. It is however, similarly difficult to parameterize the way that electrons in one material should interact across an interface with different atomic species.

The second is that the metal transport model we have simulated treats electronic behavior as diffusive. In the absence of fields, this approach is a good approximation for the thermal transport. However, many metallic systems also involve electric fields and corresponding electron drift. It seems reasonable that an implementation of the TTM with a drift–diffusion model would produce a more physical result than one with a diffusion model. In the same way that an appropriate potential will modify the phonons near the interface, a drift–diffusion TTM could modify the electronic properties near an interface by including the effects of a bias. Modeling a bias could also enable the TTM to be used for modeling Joule heating, and the only additional parameter needed would be an electron mobility.

## Data Availability

No datasets were generated or analysed during the current study.
